# Evaluation of the Application of APACHE II Combined With NIHSS Score in the Short-Term Prognosis of Acute Cerebral Hemorrhage Patient

**DOI:** 10.3389/fneur.2019.00475

**Published:** 2019-06-21

**Authors:** Xiao-Jing Zhao, Qun-Xi Li, Li-Sha Chang, Jiang Zhang, Da-Li Wang, Hai-Yan Fan, Fu-Xia Zheng, Xiu-Jie Wang

**Affiliations:** ^1^Department of Neurology, Affiliated Hospital of North China University of Science and Technology, Tangshan, China; ^2^Department of Neurosurgery, Affiliated Hospital of North China University of Science and Technology, Tangshan, China

**Keywords:** APACHE, NIHSS, acute cerebral hemorrhage, prognosis, estimated value

## Abstract

**Objective:** This study aims to evaluate the effects of combining Acute Physiology and Chronic Health Disease Classification System II (APACHE II) scores and the NIHSS score for short-term prognosis of cerebral hemorrhage patients.

**Methods:** APACHE II and NIHSS scores were respectively carried out for 189 acute cerebral hemorrhage patients who were admitted to the hospital for 24 h, and the area under ROC curve was used to measure the ability of these score systems to forecast the prognosis, in order to find the best dividing value. The discriminant analysis method should be used to carry out a comprehensive analysis of these two score methods and establish the mathematical model to provide a reasonable basis for accurately mastering these illness conditions, and its prognosis.

**Results:** The areas under the ROC curve of APACHE II and NIHSS scores in forecasting cerebral hemorrhage prognosis was 0.853 and 0.845, respectively, the dividing value was 15 and 17, respectively, and the forecasting accuracy was 77.2 and 79.9%, respectively; The forecasting accuracy of the combined discrimination model was 85.96%.

**Conclusion:** APACHE II and NIHSS scores have good forecasting value to the short-term prognosis of acute cerebral hemorrhage patients, and the combination of these two can provide a higher forecasting value.

## Introduction

Cerebral hemorrhage is a kind of frequently-occurring disease, and the objective and accurate evaluation of the illness severity of cerebral hemorrhage patients has important meaning for prognosis determination. The Acute Physiology and Chronic Health Disease Classification System II (APACHE II) is a scientific, objective and reliable score system for the intensive care unit (ICU) to evaluate the illness severity of critically ill patients and forecast the prognosis of these patients. Since its clinical application in 1985, it has become the most frequent critical disease score system in the world (1, 2). NIHSS is a comprehensive stroke scale. It is a neurological function inspection scale with 15 items and was designed in 1989 by Thmos et al. for the treatment research of acute stroke. This scale has been mainly used to evaluate the neurologic impairment degree of stroke patients. At present, there are few reports on the clinical research of APACHE II and NIHSS scores in evaluating the short-term prognosis of acute APACHE II cerebral hemorrhage patients. Therefore, this exploratory research was carried out.

## Information and Methods

### Case Selection

There were 189 cerebral hemorrhage hospitalized patients in the Neurology Department and Neurosurgery Department of North China University of Science and Technology Affiliated Hospital in 4 years. Among these patients, 117 patients were male and 72 patients were female, and the average age (x±s) of these patients was $\bar{6}$1.300±12.211 years old. Furthermore, among these 189 patients, 68 patients were conscious, 25 patients were somnolent, 12 patients were lethargic, 35 patients were in light coma status, 30 patients were in middle coma status, and 19 patients were in deep coma status. All patients entered the hospital within 3 days after onset of cerebral hemorrhage, complied with the cerebral hemorrhage diagnosis standards formulated by the Fourth National Conference on the Diagnosis of Cerebrovascular Disease Academic in 1995, and were verified by head computed tomography (CT) or (and) magnetic resonance imaging (MRI). Patients with hematologic cerebral hemorrhage and tumor cerebral hemorrhage were excluded. In the present study, patients who died within 4 weeks were considered death cases in the hospital, else these considered survival cases in the hospital (the persistent vegetative state was included). Follow-up visits and verification were carried on patients after discharge.

This study was conducted in accordance with the declaration of Helsinki. This study was conducted with approval from the Ethics Committee of the Affiliated Hospital of North China University of Science and Technology. Written informed consent was obtained from all participants.

### Evaluation Method

After all, patients were hospitalized, the APACHE II and NIHSS scores were determined for physiological parameters, laboratory inspection results, and the worst-case value of the nervous system examination and these were collected within 24 h after entering the hospital. Differences in scores between the survival group and the death group were compared. The ROC curve of these two scores for distinguishing the prognosis of patients and the area under the curve was calculated to determine the dividing value. The discriminant equation was established, taking patient prognosis as the dependent variable and APACHE II and NIHSS scores as independent variables.

### Statistical Processing

A database was established for all data using Excel, and the statistical analysis was carried out using SPSS ver. 11.5 software. The measurement data used *t*-test and used X^2^-test for the discriminant analysis. The advantages and disadvantages of the forecasting effectiveness of the scoring system were compared using the methods of Hanley and McNeil (3), and the area under the ROC curve was also compared.

## Results

**Figure 1:**
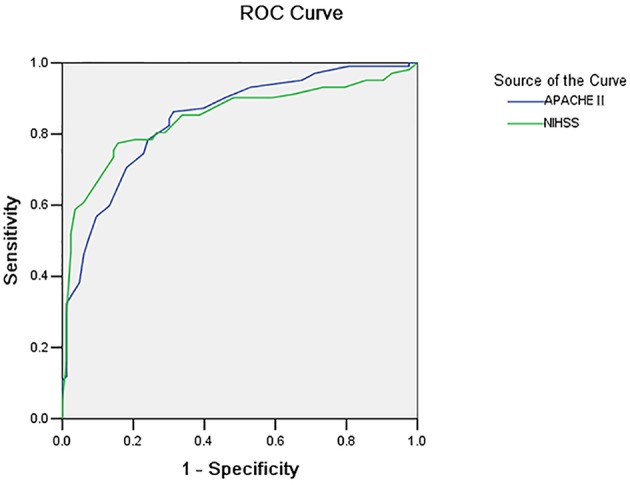
ROC curves of APACHE and NIHSS scoring.

- In comparing the value of APACHE II and NIHSS scores of patients in the survival group and death group, the value for the death group was obviously higher than that for the survival group, and the difference was statistically significant (Table 1).
- As the value of APACHE II and NIHSS scores increased, the risk of death of cerebral hemorrhage patients increased. This shows that there was an obvious dose-responsive relationship between the value of these scores and the risk of death in cerebral hemorrhage patients (Tables 2, 3).
- The area under the ROC curve for the APACHE II and NIHSS scores was 0.853 and 0.845, respectively (*p* = 0.800). The dividing value for survival and death was 15 and 17, respectively (Figure 1 and Tables 4, 5).
- The discriminant analysis of APACHE II and NIHSS scores and patient prognosis were carried out, and the gained discriminant functions are shown as follows:

$$\begin{array}{l} \text{Y0 }=\;-2.201\;+\;0.073{\text{x}}_{1}\;+\;0.223{\text{x}}_{2}\\ \text{Y1 }=\;-6.378\;+\;0.209{\text{x}}_{1}\;+\;0.363{\text{x}}_{2} \end{array}$$

Y0 and Y1 mean survival and death, respectively; x_1_ refers to the NIHSS score, while x_2_ refers to the APACHE II score, and the values are actual values. The discriminant function was used to discriminate the records of these samples, and the discriminant accuracy was 85.96%. The interactive verification was used to inspect the accuracy of the discriminant function, and the result of the accuracy was also 83.71%, which means that the discriminant function is stable (Tables 6, 7).

**Table 1:** Score comparison of cerebral hemorrhage patients in the survival group and death group.

**Scores**	**Death group**	**Survival group**	***t***
APACHEII	19.18 ± 6.44*	10.24 ± 5.73	−9.928
NIHSS	21.22 ± 8.31*	9.89 ± 6.27	−10.601

* *Comparison of patients with cerebral hemorrhage between the survival group and the death group P < 0.05*.

**Table 2:** Risk of death of the APACHE II score for the subgroups of hospitalized cerebral hemorrhage patients.

	**APACHEII scores in each subgroups**				
	**0**~****	**5**~****	**10**~****	**15**~****	**20**~****
Death in cerebral hemorrhage	1	9	12	29	52
Survival in cerebral hemorrhage	12	34	18	14	6
OR	3.176		8.000		24.857
	104.000				

*χ^2^ = 64.541, P < 0.001*.

**Table 3:** Risk of death of the NIHSS score for the subgroups of hospitalized cerebral hemorrhage patients.

**Item**	**NIHSS scores in each subgroups**					
	**0**~****	**5**~****	**10**~****	**15**~****	**20**~****	**25**~****
Death in cerebral hemorrhage	7	3	12	18	11	53
Survival in cerebral hemorrhage	17	26	19	16	3	5
OR	0.280		1.534		2.732	8.905
	25.743					

*χ^2^ = 69.594, P < 0.001*.

**Table 4:** Comparison result of the area under the ROC curve of the two scores for cerebral hemorrhage patients with the benchmark line.

**Scores**	**Area under the curve**	**Standard error**	***P***	**95%credibility interval**	
				**Low limit**	**High limit**
NIHSS	0.845	0.030	0.000	0.786	0.905
APACHE II	0.853	0.028	0.000	0.798	0.908

**Table 5:** Authenticity and predicted value of the two scores for forecasting the prognosis of cerebral hemorrhage patients.

**Scores**	**Dividing valve**	**Sensitivity**	**Specificity**	**Positive predictive value**	**Negative predictive value**	**Accuracy**
APACHEII	15	0.784	0.775	0.796	0.744	0.772
NIHSS	17	0.773	0.838	0.844	0.753	0.799

**Table 6:** Bayes discriminant analysis results of each recorded cerebral hemorrhage.

**Item**	**Treatment outcome**	**Expected discriminant result**		**Total**
		**Survival**	**Death**	
Number of cases percentage	Survival	67	13	80
	Death	12	86	98
	Survival	84.81	15.19	100
	Death	12.24	87.76	100

*result of the accuracy = 85.96%*.

**Table 7:** The cross validation result of Bayes discriminant analysis.

**Item**	**Treatment outcome**	**Expected discriminant result**		**Total**
		**Survival**	**Death**	
Number of cases percentage	Survival	66	14	80
	Death	15	83	98
	Survival	82.50	17.50	100
	Death	15.31	84.69	100

*result of the accuracy = 83.71%*.

## Discussion

Constant improvement of the accuracy of cerebral hemorrhage patient prognosis has always been one of the important subjects of clinical workers. For the critical disease score, weighing or assignment should be carried out in accordance to certain symptoms, physical signs and physiological parameters of patients, in order to quantitatively evaluate the state of the critical disease of patients, and make the illness status evaluation and prognosis forecasting of doctors scientific and objective.

The APACHE II score system comprises of three parts: acute physiology score (APS), age, and CPS. It carries out forecasting to the illness status, in accordance with the value of the APACHE II score, in which the total score ranges within 0–71, and the severity and value are positively correlated. At the same time, the APACHE II score considers the whole body physiological indicators, laboratory examination and previous physical conditions of patients. It has become the critical disease prognosis evaluation system with the widest application. The APACHE II score can also be used to evaluate the risk of death and prognosis of a stroke patient (4). Some studies have shown (5) that the APACHE II score has important reference value on cerebellar hemorrhage treatment decision-making and therapeutic evaluation, and that it is applicable for determining the illness severity of acute cerebral hemorrhage patients, as well as for prognosis forecasting (6).

NIHSS is an evaluation scale with the widest application in the world for determining the illness severity of stroke patients. It has been mainly used to evaluate the neurologic impairment degree, determine the curative effect, and evaluate the prognosis (7–10). Wei (11) considers that NIHSS can evaluate the feeling, awareness, movement, nerve and other functions of stroke patients and that its reliability is high. Some studies have shown that the NIHSS score and the average arterial pressure can effectively determine the hemorrhage risk after ischemic stroke thrombolysis (12) and that it is also applicable for studying the treatment effect of the high- pressure cerebral hemorrhage patients, and prognosis evaluation (13–15). In addition to average arterial pressure, their fluctuations over time can also significantly influence both short- and long-term prognosis of these patients (16). Foreign scholars (17, 18) have pointed out that although the NIHSS score is an effective tool to measure the severity after stroke, it has certain limitations on the dysfunction evaluation of patients.

The present study revealed that APACHE II and NIHSS scores have a close relationship with the illness severity of cerebral hemorrhage patients. Furthermore, the value of APACHE II and NIHSS scores for patients in the death group was obviously higher than that for patients in the survival group, and severity and this value were positively correlated. In addition, these findings revealed that both score methods can forecast the prognosis of cerebral hemorrhage patients, which was the same as that reported in a previous study (19). The ability of these scores to distinguish between death and survival was measured by the solution, that is, the area under the ROC curve (5). At the same time, the present study also revealed that the area under the ROC curve for APACHE II and NIHSS was 0.853 and 0.845, respectively, and that both of these were larger than the benchmark area of 0.5. This means that both of these had relatively good authenticity on the prognosis forecasting of cerebral hemorrhage patients, which was the same as that reported studies (20, 21). The method introduced by Hanley and McNeil (3) was used to compare the area under the ROC curve of these two scores, and the difference was not statistically significant. The method of Hanley and McNeil (3) uses the difference of the area under the curve between the groups, as well as the standard error of the ROC area of the two groups, to analyze the difference between the two groups. This means that the ability of these two scores, such as the solution, had no differences in distinguishing the death and survival of cerebral hemorrhage patients.

At present, these scores have no dividing value for determining survival and death, and the value reported in China ranged within 17–30 (22–25). In addition, some studies have shown that the NIHSS score had ideal specificity, sensitivity, and accuracy in the aspect of prognosis forecasting (24), and the score of 13 can be used as the dividing value for the prognosis of stroke patients. Wang et al. (26) considered the following: the area under the ROC curve of APACHE II and NIHSS scores for forecasting the prognosis of acute cerebral infarction patients was 0.835 and 0.822, respectively; the dividing value of survival or death was 12 and 10, respectively; forecasting accuracy was 74.5 and 76.9%, respectively; the forecasting accuracy of the combination of these two was 85.5%. This finding revealed that the combination of these two scores would improve the accuracy of forecasting. The present study revealed that an APACHE II score of 15 points at the first day of admission was an ideal dividing value to determine the prognosis of cerebral hemorrhage patients, and the sensitivity and specificity was 78.4 and 77.5%, respectively. The dividing value for NIHSS in forecasting cerebral hemorrhage survival and death was 17, and the sensitivity and specificity was 77.3 and 83.8%, respectively. If the score exceeds the dividing value, the illness status of the patient would be severe, the risk of death would obviously increase, and intensive care and positive treatment should be given to these patients. In the present study, it was also found that when the APACHE II value reached 30, the fatality rate was 100%, the prognosis of these patients was extremely poor, and the cost of the medical resources was extremely large, but the yield result was small.

In order to better guide clinical treatment, a cerebral infarction patient survival and death discriminant analysis model type II was established by discriminant analysis. The discriminant accuracy was 85.96%, which was superior to the prognosis determination accuracy of a single score. This means that the combination of both can improve the forecasting accuracy of the cerebral hemorrhage prognosis.

The reason why the APACHE II value for the death group was significantly higher than that for the survival group may be that some parameters of APACHE II are related to inflammation, e.g., temperature and white blood cell count and inflammation is involved in the pathological process of the acute cerebral hemorrhage. Soon after acute cerebral hemorrhage occurs, the inflammatory response is triggered by hematoma components, and inflammation together with the hematoma will worsen the damage of the brain tissue (27). In addition, it has been reported that C-reactive protein (CRP) level is positively correlated with the increased disastrous outcomes of cerebral hemorrhage, including death and vascular complications (28). The lectin pathway of complement is also reported to contribute to the pathogenesis of acute cerebral hemorrhage (29). Therefore, by assessing inflammatory biomarkers, we may learn more about the process and mechanism of acute cerebral hemorrhage injuries.

In fact, the outcome of acute cerebral hemorrhage is strongly affected by a variety of factors associated with metabolism, inflammation, cerebral perfusion and drug actions. By combining these factors, we can improve the accuracy of the current already established prognostic models. In addition to inflammation, as discussed above, studies have also shown that computed tomographic (CT) blend sign is significantly positively correlated with the CT angiography spot sign, and can predict secondary neurological deterioration after acute cerebral hemorrhage (30). Moreover, as researchers investigate more about the mechanisms of acute cerebral hemorrhage, more clinical, biochemical and imaging parameters will be found, and using the controllable parameters among all the parameters, we can improve the current prediction methods of the outcomes.

At present, most scholars tend to carry out dynamic evaluations on patients for longer consecutive days for a more effective prognosis forecasting. The present study remains in its preliminary study stage, and there was no comprehensive, systemic and large-scale clinical dynamic evaluation. Hence, this will be the direction of future studies.

## Author Contributions

X-JZ and Q-XL conceptualized and designed the study, drafted the initial manuscript, and reviewed and revised the manuscript. L-SC, JZ, D-LW, and H-YF designed the data collection instruments, collected data, carried out the initial analyses, and reviewed and revised the manuscript. F-XZ and X-JW coordinated and supervised data collection, and critically reviewed the manuscript for important intellectual content. All authors approved the final manuscript as submitted and agree to be accountable for all aspects of the work.

### Conflict of Interest Statement

The authors declare that the research was conducted in the absence of any commercial or financial relationships that could be construed as a potential conflict of interest.
